# Subjective burden on spouses of schizophrenia patients

**DOI:** 10.4103/0972-6748.62268

**Published:** 2009

**Authors:** Surekha Kumari, A. R. Singh, A. N. Verma, P. K. Verma, S. Chaudhury

**Affiliations:** Department of Psychiatric Social Work, RINPAS, Kanke, Ranchi, India; 1 Department of Clinical Psychology, RINPAS, Kanke, Ranchi, India; 2 Department of Psychiatric Social Worker, RINPAS, Kanke, Ranchi, India; 3 Department of Psychiatry, RINPAS, Kanke, Ranchi, India

**Keywords:** Burden, Schizophrenia, Spouses

## Abstract

**Background:**

There is limited information from India on subjective burden on spouses of schizophrenia patients. The aim of the present study was to assess and compare patterns of subjective burden on spouses of schizophrenia patients.

**Materials and Methods:**

The present study was conducted at the OPD level, and follow-up was done at the Ranchi Institute of Neuropsychiatry and Sciences (RINPAS) during the period May 2008 to November 2008. Tools utilized were sociodemographic data sheet, Family Burden Interview Schedule developed by Pai and R. L. Kapur (1981). The sample comprised of 50 samples of spouses (25 male and 25 female spouses of schizophrenia patients).

**Methods:**

The present study was conducted at the OPD level, and follow-up was done at the Ranchi Institute of Neuropsychiatry and Sciences (RINPAS) during the period May 2008 to November 2008. Tools utilized were sociodemographic data sheet, Family Burden Interview Schedule developed by Pai and R. L. Kapur (1981). The sample comprised of 50 samples of spouses (25 male and 25 female spouses of schizophrenia patients).

**Results:**

The findings suggest that both the groups, viz., male and female spouses of schizophrenia patients, showed moderate level of subjective burden, i.e., 13 (52%) and 15 (60%) male and female spouses, respectively, which was statistically found to be insignificant.

**Conclusion:**

No significant difference was found between male and female spouses of schizophrenia patients with regard to the level of subjective burden.

Burden of care is a complex construct that challenges simple definition, and the definitions are frequently criticized for being broad and generally negative. Frequently, burden of care is more defined by its impacts and consequences on caregivers. In addition to the emotional, psychological, physical and economic impact, the concept of ‘burden of care’ involves subtle but distressing notions such as shame, embarrassment, feelings of guilt and self-blame. The early conceptualization of ‘burden of care’ was classified into 2 distinct components — objective and subjective. Objective burden of care is meant to indicate its effects on the household, such as taking care of daily tasks; whereas subjective burden indicates the psychological and emotional impact of mental illness on family members, including feelings of grief and worry (Hoening and Hamilton, 1966). Family is an integral part of the care system for persons with a chronic mental illness. Caring for a spouse with schizophrenia is an enduring stressor and causes considerable amount of burden. Living with a schizophrenia patient can put considerable burdens and restrictions on the rest of the family. Since the mid-1950s, researches have been investigating the effects of mental illness on patients’ caregivers, regarding the impairments and emotional distress that occur as a consequence of schizophrenia. Today numerous studies document high degrees of objective and subjective burdens on relatives (Maurin and Boyd, 1990; Loukissa, 1995; Provench, 1996; Rose, 1996; Baronet, 1999). At the same time, the results of these studies raise questions as to how much a family or caregivers can provide assistance to the patient before they themselves become overburdened and require professional help. Although the burden on relatives of schizophrenia patients has been the subject of numerous studies, hardly any studies until now have focused on the live situation of spouses of schizophrenia patients. An in depth re-analysis of the exiting literature revealed that research on burden on relatives of schizophrenia patients has almost exclusive questioned parents of schizophrenia patients, and rarely the patients’ spouses (Jungbauer *et al*., 2001). This gap in research is even more surprising as there are numerous publications dealing with specific burden on spouses as caregivers of patients with other psychiatric disorders such as depression or dementia (Benazon and Coyne, 2000; Levkovitz *et al*., 2001). The spouses of schizophrenia patients have largely been neglected in previous research. One possible explanation for this might be a selection effect that occurs when recruiting study participants; for pragmatic and economic reasons, the study participants are usually recruited from among the members of self-help groups and caregivers associations, which for the most part are parents (Jones and Jones, 1994). Many researchers presuppose that only in exceptional cases are schizophrenia patients able to live in stable partnership, because of the relatively early onset of the illness and the illness-related deficits of the patient (e.g., Johnson, 2000).

A study on 307 schizophrenia patients in Leipzig, Germany, revealed that 32.9% of the subjects were married or were living with a partner (Kilian *et al*., 2001). Apparently, female patients suffering from schizophrenia have better chances of marrying or having a stable partnership than their male counterparts (Stromwall and Robinson, 1998; Hafner and Ander Heiden, 1997).

## MATERIALS AND METHODS

This study was conducted from May 2008 to Nov, 2008 at the Ranchi Institute of Neuropsychiatry and Allied Sciences (RINPAS), Kanke, Ranchi. The aim of the study was to assess subjective burden in spouses of schizophrenia patients.

The study was conducted on spouses of schizophrenia patients. The 50 subjects (25 male and 25 female patients of schizophrenia) fulfilling ICD-10 criteria chosen on the basis of purposive sampling constituted the sample, with their ages in the range of 25-60 years and 18-60 years, respectively; they were selected from the outpatient unit of RINPAS, Kanke, Ranchi. The minimum duration of patients living continuously with their respective spouses was 2 years, and their minimum educational level was up to class VIII. All the subjects were married, and only those patients coming for follow-up at the outpatient unit and who had minimum duration of illness of 2 years were included.

The subjects were administered —

1. Socio-demographic data sheet
2. Family Burden Interview Schedule (FBIS)

(Shaila Pai and R. L. Kapur, 1981).

This is a semi-structured interview schedule comprising of 24 items grouped under 6 areas: 1) financial burden, 2) disruption of routine family activities, 3) disruption of family leisure, 4) disruption of family interaction, 5) effect on physical health of others and 6) effect on mental health of others.

The burden was rated on a 3-point scale for each item, and a standard question to assess the “subjective” burden was also included in the schedule. This scale has been developed for the Indian setup, keeping in mind the socioeconomic and cultural conditions in India. The validity and reliability of the scale have been found to be satisfactory. The interrelated reliability for each item was reported to be more than 0.78 by the authors, which indicates that the present schedule is a reliable tool.

Statistical analyses were carried out using the mean, standard deviation (SD), chi-square test to determine the difference in the subjective burden between male and female spouses of schizophrenia patients.

## RESULTS

Table 1 lists socio-demographic details of the subjects. The mean age of male patients was 33.3 years and that of female patients was 37.24 years. Nearly 24% (6) of male patients and 48% (12) of female patients were illiterate. One fourth of female patients were educated up to the plus-two level; on the other hand, 40% (10) of male patients were educated up to class XII level. Surprisingly, none of the female patients was a graduate, while only 2 male patients were educated up to graduation or higher level. It is notable that 44% (11) of male patients were employed, while the majority (96%) of female patients were unemployed. The mean duration of illness was 6.32 and 7.04 years for males and females, respectively. Similarly, age at onset of illness in male patients was 26.4 years; and for female patients, it was 30.24 years. Majority of male patients, i.e., 72% (18), were found having significant past history of mental illness and were admitted to a mental hospital, the number of times admitted ranging from once to 4 times. Nearly half, i.e., 52% (13), of the female patients had no past history.

**Table 1:** Socio-demographic characteristics of patients

Variable	Male patients *n* = 25 (%)	Female patients *n* = 25 (%)
Age		
Mean	33.32	37.24
SD	7.90	8.41
Birth order		
1^st^ to 3^rd^	22 (88)	18 (72)
4^th^ to above	03 (12)	07 (28)
Total	25	25
Educational level		
Illiterate	06 (24)	12 (48)
Non-metric	07 (28)	07 (28)
Upto12^th^	10 (40)	06 (24)
Graduate and above	02 (8)	00(00)
Occupational status		
Employed	11 (44)	01 (4)
Unemployed	14 (56)	24 (96)
Duration of illness		
Mean	6.32	7.04
SD	3.37	4.81
Age at onset		
Mean	26.4	30.24
SD	7.40	7.62
Number of admissions		
1^st^-4^th^	18 (72)	12 (48)
No admission	07 (28)	13 (52)

Table 2 lists the sociodemographic details of the spouses of male and female patients with schizophrenia.

**Table 2:** Socio-demographic characteristics of spouses

Variable	Total number of spouses = 50	
	Male spouses (25) Mean/SD (%)	Female spouses (25) Mean/SD (%)
Age	43.28 (8.79)	29.4 (6.67)
Duration of stay (in years)	20.72 (13.59)	27.52 (8.41)
Educational level		
Non-matriculate	8 (32)	14 (56)
Up to class XII	13 (52)	10 (40)
Graduate and above	04 (16)	01 (4)
Occupational status		
Employed	24 (96)	07 (28)
Unemployed	01 (04)	18 (72)
Socioeconomic status		
High	02 (8)	02 (8)
Middle	06 (24)	06 (24)
Lower	17 (68)	17 (68)
Family structure		
Joint	16 (64)	15 (60)
Nuclear	09 (36)	10 (40)
Domicile		
Rural	19 (76)	18 (72)
Urban	06 (24)	07 (28)
Religion		
Hindu	17 (68)	19 (76)
Muslim	05 (20)	02 (8)
Christian	3 (12)	4 (16)

The mean ages of the spouses of male and female patients with schizophrenia were 43.28 and 29.4 years, respectively.

With regard to educational levels of male and female spouses, majority of female spouses, i.e., 56% (14), were non-matriculate, and only 4% (1) of female spouses had pursued studies up to the graduation level; whereas majority of the male spouses, i.e., 52% (13), were educated up to plus-two level only. There were 96% (24) of male spouses who were doing some kind of job, whereas 72% (18) of female spouses were unemployed.

Surprisingly, no difference could emerge between male and female spouses with regard to socioeconomic status.

A majority of male spouses, i.e., 68% (17), and a majority of female spouses, i.e., 76% (19), were following the Hindu religion.

## RESULTS AND DISCUSSION

Table 3 shows a comparison of the subjective burden between the two groups, i.e., the groups of male and female spouses. Subjective burden was categorized into 3 categories, viz., no burden, moderate burden and severe burden. The results showed that 60% (15) of spouses of female schizophrenia patients experienced moderate burden compared to 52% (13) of spouses of male schizophrenia patients experiencing moderate burden. Similarly, 40% (10) of spouses of female schizophrenia patients experienced severe burden in comparison to 36% (09) of spouses of male schizophrenia patients experiencing severe burden. Surprisingly, in 12% (03) of the spouses of male schizophrenia patients, no burden was found. The majority [52% (13)] of spouses of male schizophrenia patients experienced moderate level of burden. In the two groups, severe subjective burden was experienced by 36% of the spouses of male schizophrenia patients and 40% of the spouses of female schizophrenia patients, respectively. The difference was statistically insignificant. In this area, only 12% of the spouses of male schizophrenia patients experienced no burden, while all the remaining spouses experienced either moderate or severe burden.

**Table 3:** Comparison of subjective burden between spouses of male and female schizophrenia patients

Category	Spouses of male schizophrenia patients (*n* = 25) (%)	Spouses of female schizophrenia patients (*n* = 25) (%)	χ^2^ value	Significant level
Subjective burden				
No burden	03 (12)	00 (00)	3.2	NS
Moderate burden	13 (52)	15 (60)		
Sever burden	09 (36)	10 (40)		
Total	25	25		

## DISCUSSION

It is a well-established fact that families of patients with schizophrenia face many challenges (Hackman and Dixon, 2008), although much of the literature has focused on parents who have adult children with schizophrenia. It should be noted that 20% to 30% of patients with schizophrenia are married or are in relationships. Jungbauer *et al*. (2002) reported that spouses and partners of patients with schizophrenia experience illness burden that overlaps and extends beyond the experience of parents.

The findings of the present study clearly suggest that different levels of burden are experienced by the male and female spouses of the patients with schizophrenia, indicating that the level of burden is specific to gender. Of course, various variables have not been directly correlated to the degree of burden. Hence these issues need to be explored for obtaining more information. Creado *et al*. (2006) also found that better coping mechanisms such as problem solving can decrease the burden of illness on caregivers and may even improve the level of functioning of patients.

The findings are obvious as major mental disorders eventually lead to perceived subjective burden and disturbed interpersonal relationships. The results with regard to socio-demographic characteristics of males and females with schizophrenia show significant differences in educational and occupational status. In our sample, majority of female patients with schizophrenia had a rural background.

With regard to clinical variables, there was no significant difference between the male and female patients with schizophrenia with regard to duration of illness and age at onset.

There was no significant difference with regard to age, duration of hospital stay, educational status and type of family between the male and female spouses of patients with schizophrenia.

Among the spouses of male and female patients with schizophrenia, the results showed that there was significant difference in occupational status; 96% of spouses of female patients with schizophrenia were employed, while 28% of spouses of male patients with schizophrenia were employed.

In the present study, Family Burden Interview Schedule (Pai and Kapur, 1981) was used to assess and compare the severity of burden among the study subjects.

When scores on the subjective burden were compared between the caregiver spouses of male and female patients with schizophrenia, both the groups did not differ significantly in terms of severity of burden felt by them. Sixty percent of spouses of both the categories of patients reported moderate burden as a result of the illness of their partners.
